# A Portable Device for Simple Exosome Separation from Biological Samples

**DOI:** 10.3390/mi12101182

**Published:** 2021-09-29

**Authors:** Wenwen Chen, Yingying Xie, Yuang Chang, Yuhai Xu, Mengqian Zhao, Pengwei Deng, Jianhua Qin, Hongjing Li

**Affiliations:** 1 Dalian Institute of Chemical Physics, Chinese Academy of Sciences, Dalian 116023, China; chenwenwen@dicp.ac.cn (W.C.); xieyy@dicp.ac.cn (Y.X.); mengqianzhao@dicp.ac.cn (M.Z.); dengpengwei@dicp.ac.cn (P.D.); 2 University of Chinese Academy of Sciences, Beijing 100864, China; 3 First Affiliated Hospital of Dalian Medical University, Dalian 116011, China; changyvang_1996@163.com (Y.C.); lzn04@163.com (Y.X.); 4 CAS Centre for Excellence in Brain Science and Intelligence Technology, Chinese Academy of Sciences, Shanghai 200031, China

**Keywords:** exosome, separation, chitosan scaffold

## Abstract

Exosomes are membrane-bound nanovesicles secreted by most types of cells, which contain a series of biologically important molecules, such as miRNAs, proteins, and lipids, etc. Emerging evidence show that exosomes can affect the physiological status of cells and are involved in various pathological processes. However, due to their small size and density close to body fluids, it is challenging to separate exosomes from a small volume of biological samples in a simple manner. Herein, we propose a new strategy for isolating circulating exosomes from biological samples in a portable device. This method synergistically integrates chitosan electrostatic-adsorption, scaffold substrates, and shuttle flow to enable the highly effective capture of circulating exosomes with a recovery rate of over 80% within 20 min, which is much better than the performance of traditional ultracentrifugation (5–25%, 3 h). Besides, the isolated exosomes from samples could be lysed in situ and further subjected to RNA concentration detection and protein analysis. In particular, all the necessary procedures for exosome separation could be integrated into a single device without the need for bulky equipment. This established device is portable and easy to operate, which provides a promising platform for the study of exosome biology and clinical diagnosis.

## 1. Introduction

Exosomes are nanoscale extracellular vesicles released by almost all kinds of cells through endocytosis, fusion, and efflux [1]. They inherit a large number of substances (e.g., lipids, proteins [2,3], and nucleic acids [4]) and messages from donor cells, which play important roles in the transmission of signals and communications among cells [5]. A tremendous number of researchers described exosomes had significant effects on inflammatory processes, adaptive immunity, and tumorigenesis processes [6,7,8]. The phospholipid bilayer surrounding exosomes can protect its cargos from directly contacting with body fluids, thus as to avoid them being degraded by nuclease and protease in body fluids [9,10]. Thus, exosomes have been one of the most important biomarkers for liquid biopsy along with circulating tumor cells (CTCs) and circulating tumor deoxyribonucleic acids (ctDNAs).

Despite their potential application in clinical disease diagnosis, separating exosomes from body fluids is highly difficult due to their overlapping size range with other extracellular vesicles and similar density with body fluids. The commonly used method for exosome separation is ultracentrifugation [11,12,13]. It is suitable for the treatment of large volume samples such as culture medium, but it has some limitations in the treatment of minimal samples such as serum. The time needed for ultracentrifugation to separate exosomes from samples is about 3 hours, with a recovery rate of 5–25% [14,15]. Besides, ultracentrifugation is heavily equipment-dependent, and it is difficult to establish standardization in the clinic. Thus, a facile method without the need for bulky equipment for exosome separation from minimal biological samples is urgently necessary.

In this work, we developed a portable and simple device for circulating-exosome separation (EV-sep device) from biological samples. This device synergistically integrates chitosan electrostatic-adsorption [16], scaffold substrates, and shuttle flow to enable the highly effective capture of circulating exosomes. Using this device, exosomes can be isolated with a recovery rate of over 80% within 20 min, which is not accessible by ultracentrifugation (5–25%, 3 h). The capability of the device was also investigated using human serum, synovial fluid, and urine. This device allowed in situ exosomes lysis and further subjected to RNA and protein analysis. It may offer a promising platform for exosome clinical diagnosis and point-of-care testing.

## 2. Materials and Methods

### 2.1. Design and Fabrication of the EV-Sep Device

The EV-sep device consisted of 4 parts, including a shaker, a reactor, loading and washing buffer, and chitosan scaffolds. The rechargeable shaker (NuoMi, Jiangsu, China) was used to provide shuttle flow. The reactor was fabricated by acrylic with passageways for a mixture of samples with scaffolds, and it was bought from the Xinglin acrylic processing company (Dalian, China). The size and number of the passageways could be designed on demand. In this paper, the reactor we chose had six channels, and the volume for each channel was about 5 mL with a length of 5 cm, the width and height were both 1 cm. 

The loading and washing buffer were used to create an acid reaction atmosphere. It was 10 mM MES (2-(N-Morpholino) ethanesulfonic acid, Aladdin, Shanghai, China) with NaOH adjusting the pH to 6.0. Chitosan scaffolds were prepared by the freeze-drying method. Firstly, chitosan powder (Aladdin, 100–200 mPa·s, Shanghai, China) was dissolved in 1% acetic acid solution to form a homogeneous chitosan solution. Secondly, adding DMSO (Dimethyl sulfoxide) to the chitosan solution to control ice crystallization. After sufficient mixing, a vacuum pump was used to remove bubbles and putting the solution in a refrigerator (4 °C) for precooling. Thirdly, mixing the solution with precooled cross-linker glutaraldehyde, removing bubbles using a vacuum pump quickly, and putting the whole reaction system in a refrigerator (−20 °C) overnight. Finally, using NaBH_4_ solution to remove unreacted glutaraldehyde followed by washing twice with pure water and freeze-drying to achieve chitosan scaffolds.

### 2.2. Isolation of Exosomes on the EV-Sep Device

Exosome adsorption on the chitosan scaffold was based on electrostatic adsorption. Briefly, protonated chitosan with −NH_3_^+^ could capture exosomes, which brought anionic phosphate groups in an acid solution. In this operation, pretreated serum samples were mixed with MES at a ratio of 1:4 (sample: MES) to adjust the pH to about 6.0, while urine samples did not need to adjust the pH since they were acid inherently. Then, the samples were transformed into the reactor channels along with chitosan scaffold, following by shaking on a shaker. After exosomes were captured via electrostatic adsorption, MES was used to gently wash the chitosan scaffold to remove impurities and unabsorbed exosomes. The captured exosomes could be directly analyzed or broken down to obtain nucleic acids and proteins on the chitosan scaffold in situ. 

### 2.3. Exosome Preparation from Cell Culture Medium

The medium used for adsorption experiments was mainly derived from mouse muscle cell line C2C12, which was cultured in DMEM (Dulbecco’s Modified Eagle’s Medium) supplemented with 10% FBS and 1% penicillin-streptomycin (*v/v*), in a humidified incubator with 5% CO_2_ at 37 °C. The cell culture reagents were all from Gibco, New York, USA. Ultracentrifugation was used to obtain standard exosomes. In brief, the medium was collected and centrifuged with series of centrifugal steps at 1000 g for 10 min and 10,000 g for 30 min to discard cell debris and large microvesicles. Then the substances with sizes above 220 nm were filtered using a 220 nm filter membrane (Millex, Atlanta, GA, USA). The filtrate was ultracentrifuged at the speed of 120,000 g to precipitate exosomes, following by PBS washing and resuspending. The resulting exosome samples were stored at −80 °C until use. 

### 2.4. Clinical Samples Collection and Pretreatment

Clinical blood, synovial fluid, and urine samples were collected from the First Affiliated Hospital of Dalian Medical University according to the protocols approved by the institutional review committee (PJ-KY-2019-96(X)). They were stored at −80 °C until use, and repeated freezing and thawing must be avoided. Pretreatment of samples was the same as that of the culture medium. Cells and cell fragments were removed by centrifuging at 1000g for 10 min at 4 °C. Collected supernatant into a new tube and centrifugated with 10,000 g for 30 min at 4 °C to remove larger microvesicles. Finally, the collected supernatant was filtrated through a commercial filter with an aperture of 220 nm.

### 2.5. Quantitative Analysis of Exosomes

The quantitative protein method is one of the common quantitative methods of exosomes. In this paper, the concentration of exosomes was measured by a fluorometer (Qubit 3.0, Waltham, MA, USA) and a protein assay kit (Invitrogen, Waltham, MA, USA). The standard curve was set up firstly using different concentrations (0, 200, 400 μg/mL) of standard protein samples. The concentration of exosomes was quantified using the relative protein content. The capture efficiency was calculated by the following equation:

$$\eta =\frac{\eta_{0}-\eta_{1}}{\eta_{0}}$$

where $\eta_{0}$ denotes the relative concentration of exosomes of original samples, $\eta_{1}$ denotes the relative concentration of exosome after capture.

### 2.6. Scanning Electron Microscope Imaging 

The scanning electron images for the material structure were obtained by coating freeze-dried chitosan scaffold with gold using a sputter coater (KYKY Technology Co. LTD., Beijing, China) and imaging with a scanning electron microscope (SEM) (Jeol Ltd., Akishima-Shi, Japan).

The structure of exosomes was observed by a scanning electron microscope with higher resolution. After exosomes were absorbed on the surface of the chitosan scaffold, using 4% paraformaldehyde (Tianjin Damao, Tianjin, China) in PBS to fix samples for 15 min and washing twice with PBS. Then dehydrating samples in a series of alcohol solutions (25%, 50%, 75%, 90%, 95%, 100% alcohol in PBS), followed by drying at ambient temperature for 30 min and coating with platinum using a sputter coater (Leica ACE200, Wetzlar, Germany) before imaging with a field emission scanning electron microscope (FESEM) (JSM-7800F, Akishima-Shi, Japan).

### 2.7. Microscope Images and Photos

The microscope images were obtained by microscope (Leica, Wetzlar, Germany), and the photos of the device and scaffold were taken by a mobile phone (Xiaomi, Beijing, China).

### 2.8. Aperture and Porosity Measurement

Image J was used for aperture and porosity measurement. Three microscopy images of different scaffolds were chosen firstly, then we set the scale and turned the type of image into 8-bit. For the measure of aperture, 10 complete circular holes on each image were randomly selected, and the distance between the farthest 2 points in a circular hole was measured. For the measure of porosity, pore area, and total area could be measured by Image J, and the porosity was equal to pore area/total area.

### 2.9. RNA Quantification

After exosomes were captured on the chitosan scaffold, nucleic acids were extracted with Trizol (Takara, Mountain View, CA, USA) in situ. After being resuspended with DEPC (Diethyl pyrocarbonate) water, an ultramicro spectrophotometer (nanophotometer N50, IMPLEN, Munich, Germany) was used to measure the total RNA concentration of the samples.

### 2.10. Western Blot

Exosomes separated by EV-sep device and ultracentrifuge were lysed using protein lysis buffer (1% protease inhibitor and 1% rmsf in RIPA). SDS-PAGE was used to separated protein lysates. Then transferring the gels onto NC membranes and immunoblotting with antibodies against CD63 and CD9 (Abcam, Cambridge, UK), following by horseradish peroxidase-conjugated secondary antibodies incubation. The method used for immunodetection was chemiluminescence. All reagents for Western blot were bought from Beyotime, Shanghai, China.

### 2.11. Particle Size Analysis

Particle size was analyzed using a nano-laser particle detector (Zetasizer Nano, Malvern, UK).

### 2.12. Statistical Analysis

All measurements were performed in triplicate, and the data were presented as mean ± S.D. Statistical significance analysis was performed using Student’s *t*-test for 2 groups. The p-value less than 0.05 was considered statistically significant.

## 3. Results and Discussion

### 3.1. Design and Operation of the EV-Sep Device

**Figure 1:**
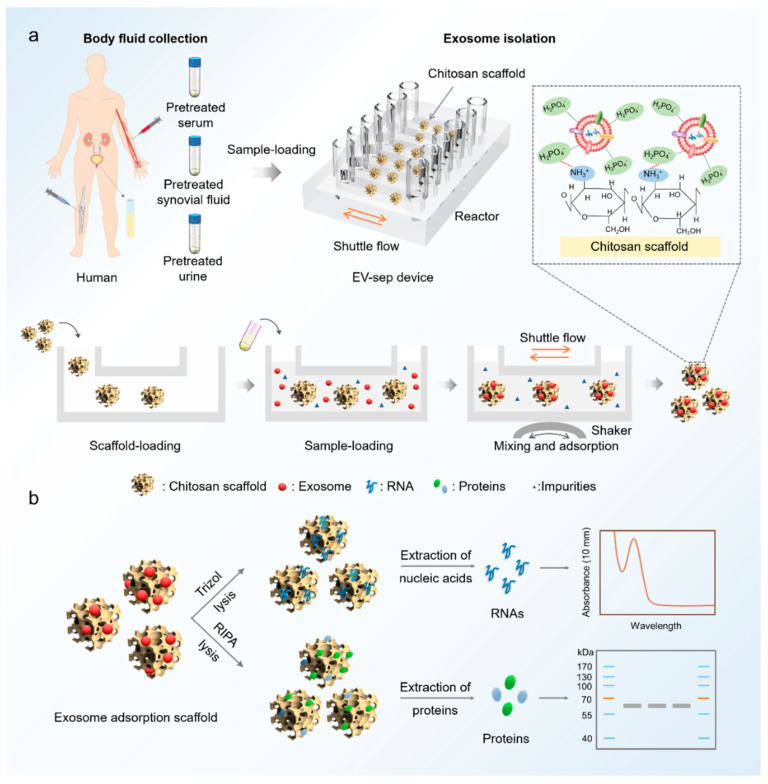
The illustration of exosome isolation from body fluids using EV-sep device. (**a**) Schematic diagram of the device for exosome isolation from serum and urine of human. In an acidic environment, −NH_3_^+^ on chitosan can combine with anionic phosphate groups on phospholipid bilayer of exosomes. (**b**) The exosomes captured by the EV-sep device can be lysed online for nucleic acids or proteins analysis subsequently.

This EV-sep device synergistically integrates chitosan electrostatic-adsorption, scaffold substrates, and shuttle flow to enable the highly effective capture of circulating exosomes. As shown in Figure 1a, body fluids such as urine, synovial fluid, and serum can combine with the chitosan scaffolds in the reactor for exosome separation. The chitosan scaffold has a large number of amino groups, which are positively charged in the acidic solution on its surface. Since the phosphate group of phospholipid bilayer on exosomes has negative charges, the chitosan can capture the exosomes based on the electrostatic adsorption in the acidic solution. Meanwhile, the captured exosomes can be lysed in situ to extract RNAs and proteins for molecular analysis (Figure 1b). The shaker and reactor are used to increase the mixing and contacts of liquid samples with micro-structured substrates to enhance the capture efficiency. 

**Figure 2:**
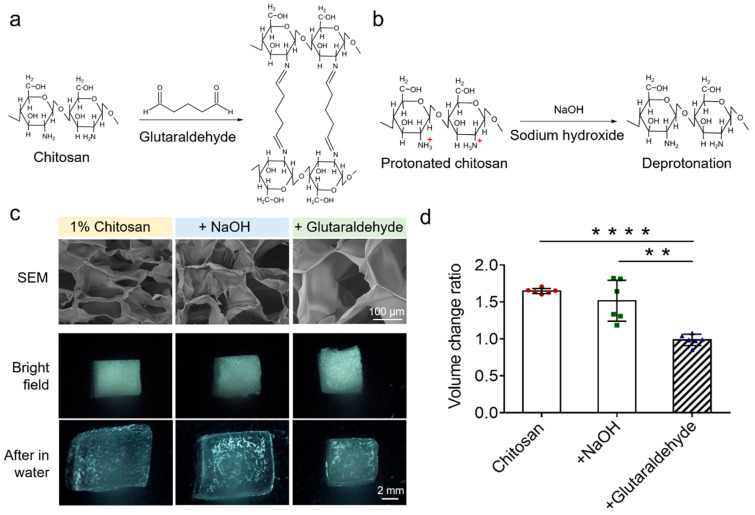
Selection of reaction system of the chitosan scaffold. (**a**) Chemical reaction equation of chitosan cross-linking by using glutaraldehyde. (**b**) Chemical reaction equation of chitosan cross-linking by using sodium hydroxide. (**c**) Scanning electron microscopy and optical microscopy images of chitosan scaffolds in different conditions, including without cross-linking, cross-linking by sodium hydroxide, and cross-linking by glutaraldehyde. The images of chitosan scaffolds after being soaked in water were also provided. (**d**) Volume change ratio of chitosan scaffolds before and after being soaked in water. The data were present as mean ± S.D. *** *p* < 0.0001 and * *p* < 0.005 by two-sided paired Student’s *t* test.

Based on the electrostatic adsorption principle of the EV-sep device, the property of the chitosan scaffold is an important factor affecting exosome isolations. Glutaraldehyde and NaOH are two of the most commonly used cross-linkers for chitosan cross-linking [17,18]. The principle for glutaraldehyde and chitosan reaction is aldimine condensation (Figure 2a), which is a covalent cross-linking process of compounds with aldehyde and amino groups by condensing aldehyde groups and amino groups into Schiff bases [19]. The principle for NaOH is deprotonation (Figure 2b). The solvent of chitosan is 1% acetic acid, in which the amino groups of chitosan can be protonated into ammonia ions. While in the alkaline environment, chitosan will be deprotonated and precipitated.

In order to ensure the contact between samples and substrates, the scaffold substrates should be stable and have a large specific surface area. We investigated the morphology and stability of chitosan scaffolds fabricated via the two most commonly used methods after being soaked in water. As shown in Figure 2c, a scanning electron microscope (SEM) was used to observe precise structures of freeze-dried chitosan without cross-linking and cross-linking with NaOH or glutaraldehyde. The SEM results show that freeze-dried chitosan in all three conditions had porous structures, while the surface of chitosan cross-linked by glutaraldehyde was smoother. In order to investigate their stability, we cut chitosan scaffolds into small pieces and put them in water. The results show that chitosan without cross-linking and cross-linked by NaOH began to swell in water with the volume change ratio of around 1.5, while there was no obvious volume change for chitosan cross-linked by glutaraldehyde (Figure 2d). These results indicate that the shape of pores in chitosan without cross-linking and cross-linked by NaOH would change after being soaked in water. It is worth noting that these two kinds of pieces would fracture after experiencing shuttle flows on the shaker, while chitosan cross-linked by glutaraldehyde did not. Collectively, glutaraldehyde was chosen as the cross-linker for chitosan.

### 3.2. Optimizations of Chitosan Scaffolds of the EV-Sep Device

**Figure 3:**
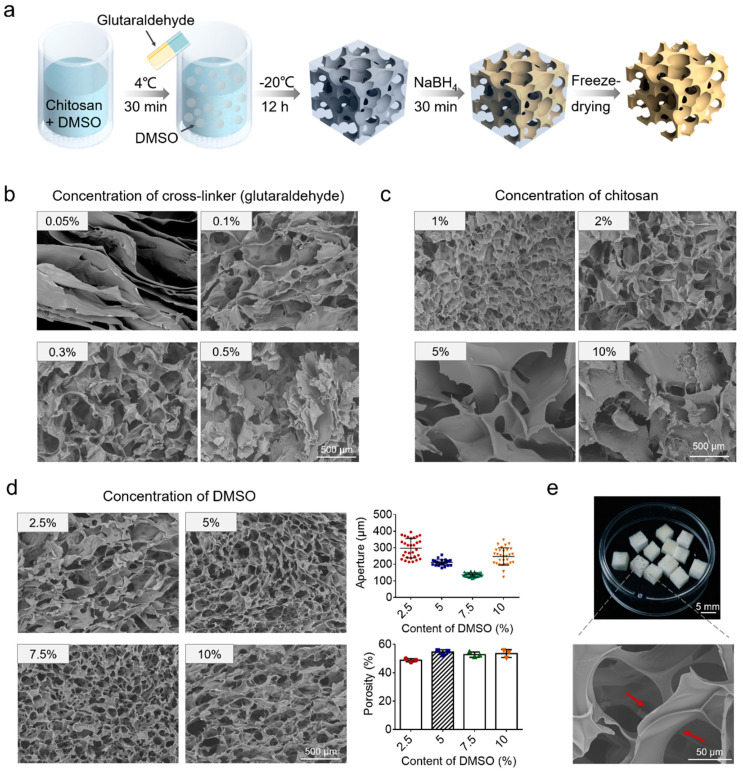
Optimization of the chitosan scaffold. (**a**) A general workflow was developed for the fabrication of the chitosan scaffold. (**b**) SEM images of chitosan scaffold fabricated with different concentrations of cross-linker glutaraldehyde. The concentration of chitosan was fixed at 2%. (**c**) SEM images of chitosan scaffold fabricated with different concentrations of chitosan. The concentration of glutaraldehyde was fixed at 0.3%. (**d**) SEM images, aperture, and porosity of chitosan scaffold fabricated with different concentrations of DMSO. The concentration of glutaraldehyde and the concentration of chitosan were fixed at 0.3% and 2%, respectively. (**e**) Photo and enlarged view of the optimized chitosan scaffold. Red arrows indicate the pores in the chitosan scaffold are connected to each other.

The chitosan scaffold is the core part of the EV-sep device. It should have a large specific surface area and controllable pores. In order to optimize the performance of EV-sep device for exosomes isolation, the chitosan scaffold was optimized first from three aspects, including the concentration of glutaraldehyde, concentration of chitosan, and the addition of DMSO. The whole schedule is shown in Figure 3a. DMSO has been shown to have the capacity to fine-tune the ice crystal formation during the cooling process, therefore, conveniently controlling the pore size of the free-dried scaffolds [20]. Then, precooled cross-linker glutaraldehyde was added into chitosan solution and mixed evenly. After putting the whole system at −20 °C overnight to make the cross-linking complete, NaBH_4_ was used to remove the unreacted cross-linker, followed by washing twice with water. Dry chitosan scaffolds can be obtained through freeze-drying. 

The effects of different concentrations of cross-linker glutaraldehyde were investigated firstly. The concentration of chitosan was 2% (weight/volume ratio). According to the molecular formula of glutaraldehyde cross-linking chitosan reaction, the molecular weight ratio of glutaraldehyde and chitosan was about 1:4, which means when the concentration of chitosan was 2%, the concentration of glutaraldehyde should not exceed 0.5% to make sure there would be amino groups left to function. Therefore, we chose glutaraldehyde concentrations of 0.05%, 0.1%, 0.3%, and 0.5% to test. Results are shown in Figure 3b. When the concentration of glutaraldehyde was lower than 0.1%, the scaffold structures would be laminated rather than porous. With the increase of concentrations of cross-linker, the pore structure became more and more obvious. However, when the concentration was higher than 0.5%, pores began to somewhat collapse due to excessive cross-linking. Besides, when the concentration of the cross-linker was too high, a large number of amino groups on the surface of chitosan will be reacted, which is not conducive to the follow-up experiments. Therefore, we chose 0.3% as the final concentration of glutaraldehyde. 

Based on the reaction formula, we optimized the concentration of chitosan subsequently. We chose chitosan concentrations of 1%, 2%, 5%, and 10% to test, and the concentration of glutaraldehyde was fixed at 0.3%. The results are shown in Figure 3c. When the concentration of chitosan was lower than 1%, there were a large number of fine fibers with almost no complete pore structure in the chitosan scaffold. When the concentration of chitosan was 2%, we could see that there were interconnected pores in the chitosan scaffold. When the concentration of chitosan was higher than 5%, the wall between two pores was too thick, and the pores were almost disconnected. Taking all the factors into consideration, we chose 2% as the concentration of chitosan. 

In order to investigate the effects of DMSO on the pore structure of chitosan scaffolds, we fabricated chitosan scaffolds with different concentrations of DMSO: 2.5%, 5%, 7.5%, 10%. The concentration of glutaraldehyde and the concentration of chitosan were fixed at 0.3% and 2%, respectively. Image J was used to characterize their porosity and pore size distribution. The results are shown in Figure 3d. When the concentration of DMSO increased from 2.5% to 7.5%, the uniformity of pores increased with the increase of concentration of DMSO, while the pore size decreased. On the contrary, when the concentration of DMSO was larger than 7.5%, the uniformity of pores decreased with the increase of concentration of DMSO, while the pore size increased. The aperture of pores was relatively uniform when the concentrations of DMSO were 5% (200 μm) and 7.5% (150 μm). What is more, we also calculated the porosity of these scaffolds. The results show that when the concentration of DMSO was 5%, the porosity of the scaffold appeared to be the largest. On the whole, however, there was no significant difference in porosity among different concentrations of DMSO. A larger aperture will benefit the contact between samples and substances. Considering the above factors, we finally chose 5% as the final concentration of DMSO. The prepared chitosan scaffold is shown in Figure 3e. It can be seen that the chitosan scaffold has obvious through-hole structures, which will be beneficial for liquid exchanges.

### 3.3. Characterizations of the EV-Sep Device for Exosome Capture

**Figure 4:**
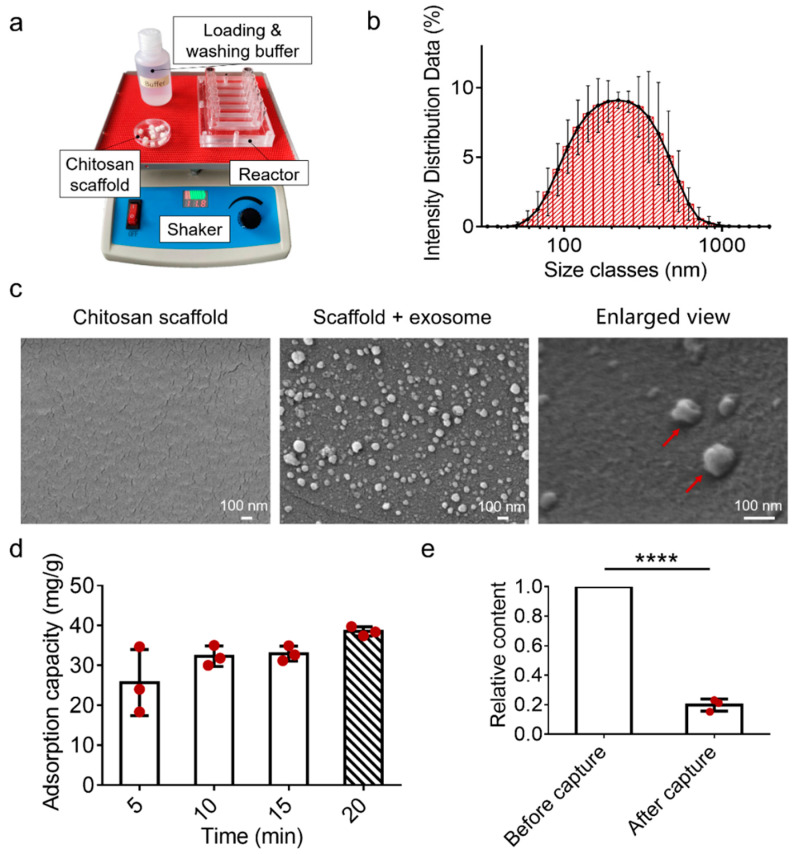
Characterization of the EV-sep device for exosome capture. (**a**) The device is composed of chitosan scaffolds, loading and washing buffer, a reactor, and a shaker, which provides shuttle flows. (**b**) Particle size analysis of standard exosome samples. (**c**) SEM images of chitosan scaffold before and after exosome capture. Red arrows show that particles with a size about 100nm and traditional exosome structure are absorbed on the surface of scaffolds. (**d**) Adsorption capacity of the chitosan scaffold for exosome capture. (**e**) The relative content of exosomes before and after adsorbed by the chitosan scaffold device. The data were present as mean ± S.D, *n* = 3. **** *p* < 0.0001 by two-sided paired Student’s *t* test.

In order to verify the performance of the EV-sep device for exosome isolation, exosomes obtained by ultracentrifugation were used to test and optimize the performance of the device. While chitosan will be protonated in an acidic solution to bring positive charges, exosomes that are negatively charged on the surface can be adsorbed on the surface of chitosan substances. The device consisted of chitosan scaffolds, a rechargeable shaker, a reactor, and loading and washing buffer (Figure 4a). Exosome samples obtained by ultracentrifugation were identified by nanoparticle size analyzer. As shown in Figure 4b, the result indicated that exosomes obtained by ultracentrifugation were about 200 nm in diameter, and the distribution was relatively uniform. 

SEM was used to characterize the adsorption of exosomes on the EV-sep device. As shown in Figure 4c, the surface of the chitosan scaffold without exosome adsorption was smooth, while there were a lot of white small particles with the typical bowl-like structure of exosomes on the surface of the chitosan scaffold after exosome adsorptions. The results confirmed that exosomes could be captured on the EV-sep device. 

To assess the exosome-capture efficiency of the device, the exosome adsorption capacity of the EV-sep device was investigated. The shaker we used had a fixed swing angle of 15° and an adjustable speed from 15–80 rpm/min. Considering that the magnitude of shuttle flow might also influence the capture efficiency of exosomes, we fixed the speed of the shaker to 30 rpm/min and optimized capture time and capability on this base. The exosomes were quantified using Qubit and its kit. Excessive standard exosome samples were used to study the effect of capture time on the adsorption capacity. The results showed that the adsorption capacity of exosome per gram of scaffold could reach 32 mg/g within 10 min, then the capture capacity changed little with time. At 20 min, the adsorption capability of the scaffold reached 40 mg exosomes per gram (Figure 4d). We also studied the recovery rate of exosome standard samples isolated by this EV-sep device in 20 min. The results are shown in Figure 4e. Because the original concentration of exosomes obtained in each experiment was not completely consistent, we defined the original concentration of exosomes as 1 ($\eta_{0}$). The relative concentration of exosomes after capture ($\eta_{1}$) was normalized by their own original concentration. The capture efficiency could reach up to 80%.

### 3.4. Performance of EV-Sep Device for Exosome Separation from Clinical Samples

In order to evaluate the performance of the EV-sep device, we used pretreated serum (1 mL), synovial fluid (1 mL), and urine (5 mL) to validate our method by comparing it with ultracentrifugation. The amount of chitosan we used for each sample was 0.005 g. The results were compared from three aspects, including operation time, protein extracted efficiency, and nucleic acid extracted rate. 

**Figure 5:**
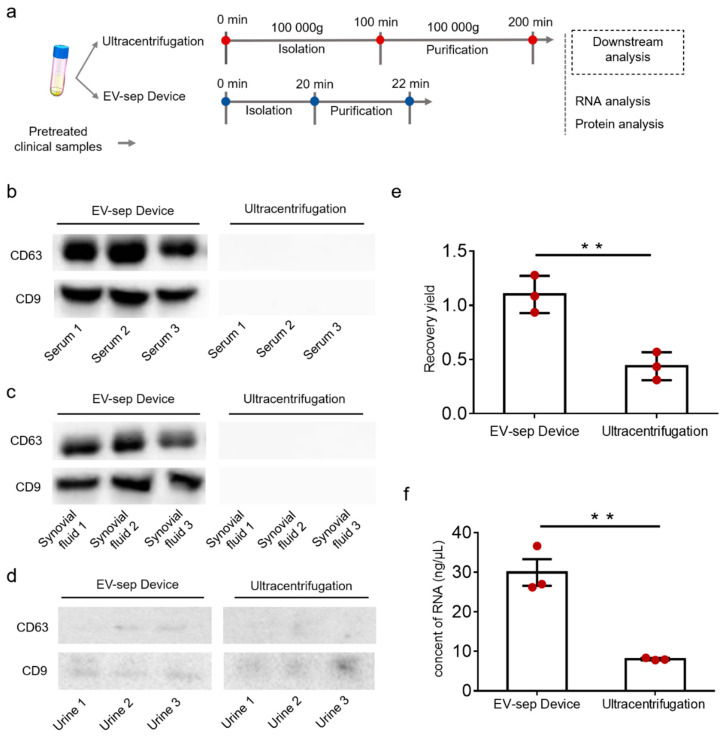
Comparison of exosome isolation in the EV-sep device and ultracentrifugation. (**a**) Workflow of the exosome isolation process of EV-sep device and ultracentrifugation. (**b**) Expression of exosome markers (CD9 and CD63) in serum exosomes isolated by EV-sep device and ultracentrifugation. (**c**) Expression of exosome markers (CD9 and CD63) in synovial fluid exosomes isolated by EV-sep device and ultracentrifugation. (**d**) Expression of exosome makers (CD9 and CD63) in urine exosomes isolated by EV-sep device and ultracentrifugation. (**e**) The recovery ratio of total RNA in serum exosomes isolated by EV-sep device and ultracentrifugation. The results were normalized to pretreated serum. (**f**) The concentration of total RNA in urine exosomes isolated by EV-sep device and ultracentrifugation. All experiments were performed in triplicate and the data were shown as mean ± S.D. ** *p* < 0.005 by two-sided paired Student’s *t* test.

As shown in Figure 5a, separation of exosomes from pretreated samples using ultracentrifugation or the EV-sep device both had two steps: isolation and purification. The time needed for ultracentrifugation to separate exosomes from pretreated body fluids was more than three hours, while it only needed less than half an hour for the EV-sep device. Besides, ultracentrifugation is equipment-dependent, while the EV-sep device is only about 2 kg in weight with a small footprint. Cooperated with rechargeable shaker, this device might further expand its applications. 

As for the comparison of proteins, CD9, CD63, and CD81 are the main proteins that are usually used to identify exosomes from other proteins. Here, we selected CD9 and CD63 to quantify exosome-related proteins and analyzed them using Western blot. For the separation of pretreated serum and synovial fluid, the results demonstrated that the mass of CD9 and CD63 proteins extracted through the EV-sep device was higher than those isolated using ultracentrifugation, revealing that the isolation performance of EV-sep device was better than ultracentrifugation in the exosome separation of pretreated serum (Figure 5b,c). For the separation of pretreated urine, as shown in Figure 5d, both methods could separate exosomes from urine, but the quantities were both small. This might be attributed to the fact that the concentration of exosomes in urine is much smaller than that in serum.

The nucleic acids were also extracted from the pretreated clinical samples, EV-sep device separated samples, and ultracentrifugation separated samples, respectively, by the Trizol method, and the concentrations of the total RNA were detected using a nanophotometer. Since the protein results of synovial fluid and serum are similar, we chose serum as the representative. For the separation of pretreated serum, both the EV-sep device and ultracentrifugation were normalized to the control group accordingly. The results showed that the recovery ratio of total RNA of EV-sep device was 1.10 ± 0.14, while the recovery ratio using ultracentrifugation was only 0.44 ± 0.11 (Figure 5e). For the separation of urine, we showed the concentration of RNA rather than the recovery rate since it was impossible to extract nucleic acid from urine directly. As shown in Figure 5f, the RNA concentration extracted by the EV-sep device could reach up to 30 ng/μL, while the ultracentrifugation could only reach to 10 ng/μL. These results indicated that the EV-sep device exhibited advantages over traditional ultracentrifugation in the isolation of trace clinical biological samples in terms of cost, portability, accessibility, and efficiency.

## 4. Conclusions

In this work, we proposed a portable and simple device for the isolation of exosomes from biological samples such as serum and urine. This EV-sep device can capture exosomes from biological samples with a recovery rate of over 80% within 20 min, which is not achievable for the traditional ultracentrifugation method. We further used the EV-sep device to isolate exosomes from serum, synovial fluid, and urine samples, followed by RNAs and proteins analysis. The results show that this device allowed in situ exosomes lysis and was further subjected to RNA and protein analysis, resulting in some extent of higher recovery yields of proteins and RNAs comparing with ultracentrifugation.

The novelties of this method lie in that this device is portable and easy to popularize with a weight of only about 2 kg. It offers a quick (within 20 min) approach to efficiently isolating exosomes from minimal biological samples; the chitosan electrostatic-adsorption eases the damages to exosomes during isolations. Besides, this device also has some limitations. Free proteins and free nucleic acids may also be absorbed on the surface of the chitosan scaffold. We are now trying to add pretreatment steps to remove free proteins and free nucleic acids from the samples in order to obtain more pure exosomes. The established approach may provide a new exosome separation choice for exosome studies, especially in the application of exosome isolation from minimal clinical samples and the exosome biology and clinical diagnosis.
